# Supplementary material for: Exploring the role of human resource development functions on crisis management: The case of Dubai-UAE during Covid-19 crisis
Source: PLoS One. 2022 Mar 8;17(3):e0263034. doi: 10.1371/journal.pone.0263034 (PMC8903275; doi:10.1371/journal.pone.0263034)
Supplement: Attachment — Submitted filename: Manuscript Number PONE-D-21-34065 opinion.docx [file pone.0263034.s002.docx]

❏ Identify the research question and key claims

**Study Problem:**

Many organisations face failures while an organisational crisis happens, whereas some organisations emerge as successful leaders amidst the crisis period.

**Introduction:**

1.Human Resource Development (HRD) is defined as “the organised learning venture displayed within an organisation to enhance personal growth and professional performance of the employees”. HRD aims to improve organisational efficiency to enhance the individual, the job and the organisation (Ulrich, 2020).

2. The recent incidence crisis (Covid-19) can be internal business conflicts or external affairs like trade policy, geopolitical issues and others. The organisations must develop a crisis preparedness approach that must include almost all the possible actors for a business crisis such as emergency response approach, communication crisis, IT issues and significant others is; however, businesses worldwide have faced different types of crises, which have disrupted their business model.

**Abstract:**

1.This study is trying to test the impact of certain HRD functions on the effectiveness of crisis management.

2.To evaluate the importance of HRD during a crisis period by examining the existing literature.

**opinion:**

The employees have been Conducted for formal or informal induction training? The employees have completed some crisis management planning projects or assessments, such as a questionnaire? ......and others. If the employees answer to any of the above questions is yes, then they have been involved in some forms of human resource development functions on crisis management. That is the role of Human Resource Development functions on crisis management should be explored? It means to explore the employees’ a talent suitable for crisis management position. The result can support the organization regards employee education, training and development as an important part of its crisis management strategy. As a set of systematic and planned activities that organizations provide employees face with crisis management opportunities to learn necessary skills to meet current and future work needs.

❏ Think about context and related literature

1. These steps establish and reinforce an organizational priority for crisis management thinking and action which is integrated through early warning signs, preparation and prevention stage, damage containment stage, activity recovery phase and learning stage.
2. The six hypothesis of HRD functions were disscused to have a direct effect on CM, it can be said that training, leadership, organisational strategy, organisational structure, Job Values and uniqueness and organisational culture.
3. It means those have a positive and significant impacts on crisis management upon an increase of those factors, crisis management effectivenessd can be increased.

**opinion:**

As far as the challenges of enterprises and institutions are concerned, the prevention and control of the virus is only the beginning of the crisis. As the virus continues to raging and the impact of the crisis is arduous after the resumption of work, what methods and strategies can be applied to the hidden opportunities that are not obvious in the crisis?

❏ Look at the figures and tables. Are they clear? Do they represent what the study is about?

1. Structural Model and Hypothesis Testing show “ Bootstrapping was performed to provide.., and the 500 **samples**. Table 1 total sample 300(or 300X500=150000); Chin(2010) and Hairetal(2011) suggest the numbers add up to 5000.
2. The References vanished without trace in “Table 2: Sources of statements used in the survey” for Paul C. Light(2009), Light(2007), Sumic and Labas (2019), Mousavi, et al. (2015)
3. The title vanished without trace on Table 4.
4. There is a slight difference between Table 5 and Figure (2) The Path Analysis Results about EWS1 (0.436-0.836), EWS2 (0.621-0.821), EWS3 (0.64-0.74) and LS1 (0.822-0.722).
5. Context” All these results are summarized in Table 5” should be Table 6 and the title” Path Coefficient of the Research Hypotheses “ vanished without trace on Table 6 .
6. It should put in down for the title” Figure 1: Conceptual Framework”.

**opinion:**

Concerning the fact that test results of hypothesis four out of six HR functions were found to have a direct effect on CM, it can be said that training, leadership, organisational strategy and organisational culture have a positive and significant impact on crisis management effectiveness. It means also that upon an increase of the other factors, effective crisis management can be increased.

❏ Examine the results. Are they supported by the data?

**opinion:**

The results are supported by the data. But what happen can be discussed for the result turned out “Not Supported” for “Organisation structure -> Crisis Management”. Because the item OST3” The organisation experiences effective communication among the team members through this organisational structure” has loading for 0.902, and in the textbook, crisis Management is called as crisis communication management. Strengthening information disclosure, communication with the public, striving for public understanding and support are the basic countermeasures for crisis management.

❏ Read the conclusions. Do they make sense?

**opinion:**

It can be made more senses according to study aim and objectives.

❏ Check the methods. Are they appropriate and reproducible?

**opinion:**

AS the context and related literature said” the Covid-19 pandemic has affected both the professional and personal lives of the people (Nadworny, 2020). The worldwide travel restrictions, closed stores and restaurants, social distancing, lockdown and emerging recession have adversely affected the employers and the employees.” In general, efforts tend to flow outward from a staff function, to sharing resources among crisis management team members, to thinking and preparing for crisis management throughout the entire organization. Crisis management efforts cut across organizational boundaries. Procedures for dealing with external stakeholders (vendors, customers, competitors, and the like) are eventually folded into crisis management plans and representatives of such stakeholder groups are informed of their roles and may even take part in the organization's crisis simulations. The case of Dubai-UAE during Covid-19 crisis is hard word according to study aim and objectives said “ To analyse the effective measures taken by the UAE private and public sectors to maintain workforce welfare during a crisis period like Covid-19 pandemic through evaluating existing journals and articles”.
